# Dissociating certainty and uncertainty for passengers of autonomous vehicles: evidence against a perfect inverse relationship

**DOI:** 10.3389/fpsyg.2026.1917625

**Published:** 2026-10-23

**Authors:** Mervyn Franssen, Erik D. van der Spek, Marieke H. Martens

**Affiliations:** 1 Department of Industrial Design, Eindhoven University of Technology, Eindhoven, Netherlands; 2 Integrated Vehicle Safety, TNO, Helmond, Netherlands

**Keywords:** automated driving, certainty, crowdsourcing, passenger, uncertainty

## Abstract

This study examines whether psychological uncertainty can be understood as the inverse of certainty in the context of fully automated vehicle (AV) passengers. Using a large-scale crowd-sourced experiment (*N* = 1,437 participants from 84 countries), participants viewed 3D simulated driving scenarios and rated their perceived certainty and uncertainty regarding an upcoming lane change. A linear mixed-effects model was used to analyze the relationship while controlling for repeated measures induced biases and individual variability. Results show a significant negative relationship between certainty and uncertainty (*B* = −0.320, *p* < 0.01), indicating that higher certainty is associated with lower uncertainty. However, the magnitude of this relationship deviates strongly from a perfect inverse (*B* = −1), demonstrating that the constructs are not simply opposites. Instead, certainty and uncertainty appear to function as partially independent psychological dimensions that can coexist. These findings challenge the common assumption underlying bipolar measurement scales and support the use of unipolar approaches when assessing subjective uncertainty. The results have implications for human-automation interaction design, suggesting that reducing uncertainty does not necessarily guarantee increased certainty. A more nuanced understanding of both constructs can contribute to improving trust, user experience, and acceptance of automated driving systems.

## 1 Introduction

Automated vehicles are expected to improve road safety (Jeong et al., 2017; Verberne et al., 2012), enhance comfort (Milakis et al., 2017), reduce congestion (Singh et al., 2025), and enable engagement in non-driving activities (Hancock et al., 2020). These developments are particularly important given that traffic-related fatalities remain high worldwide (WHO, 2023; Bougna et al., 2022). Nevertheless, public acceptance remains limited due to concerns about trust, safety, and responsibility (Kyriakidis et al., 2015; Othman, 2021; Rezaei and Caulfield, 2020). Designing intuitive and transparent systems is therefore crucial (Flemisch et al., 2012; Hoc et al., 2009), because users often struggle to form accurate mental models of the capabilities and limitations of an automated vehicle (Seppelt and Lee, 2007; Nasser et al., 2025), which can increase uncertainty. Uncertainty is frequently experienced in the context of automated driving (Franssen et al., 2024a). Passengers may feel uncertain about the behavior, reliability, and intentions of fully automated vehicles (Beller et al., 2013), which may undermine the anticipated benefits of comfort and relaxation. More broadly, uncertainty has been described as the difficulty of predicting outcomes in dynamic environments (Hsu et al., 2005; Huettel et al., 2005; Volz et al., 2003), or as a cognitive-emotional state in which individuals cannot determine which of several possible outcomes will occur (Penrod, 2001; Greco and Roger, 2003; Bar-Anan et al., 2009; FeldmanHall and Shenhav, 2019; Salah et al., 2023; Alquist and Baumeister, 2024).

Uncertainty often arises from limited knowledge and information in interactions between humans and automated systems (Meissner et al., 2020; Lingam et al., 2025a). Understanding psychological uncertainty is therefore essential for designing clear and intuitive interactions with automated vehicles. In this study, we focus on psychological uncertainty, as it captures a broad mental experience encompassing cognitive, emotional, motivational, and behavioral aspects (Morriss et al., 2022; Franssen et al., 2024b; Anderson et al., 2019). This distinguishes it from cognitive uncertainty, which primarily concerns reasoning and mental representations (Pouget et al., 2016; Meyniel et al., 2015). Psychological uncertainty can negatively influence trust (Lingam et al., 2026) and reduce the acceptance of automated technologies (Lee and See, 2004). Although trust is closely linked to the acceptance of automated vehicles (Choi and Ji, 2015; Cysneiros et al., 2018; Detjen et al., 2021; Ghazizadeh et al., 2012; Hoff and Bashir, 2015; Verberne et al., 2012; Walker et al., 2023; Wilson et al., 2020), trust and uncertainty are conceptually distinct (Lewis and Weigert, 1985; Hashidate, 2025; Schilke et al., 2026). Uncertainty arises from limited knowledge and perceived complexity, whereas trust reflects the willingness to accept vulnerability despite this uncertainty (Luhmann, 1968, 1979; Lewis and Weigert, 1985; Hashidate, 2025; Schilke et al., 2026). The literature review of Franssen et al. (2026) on psychological uncertainty presented a single definition for psychological uncertainty: “*Psychological uncertainty is the mental experience of a lack of knowledge about the past, present, or future, which makes it impossible to accurately predict outcomes or determine which of multiple possibilities will be true, and which in turn shapes emotional responses and behavioral output.”* This still raises a fundamental question: can uncertainty be understood as the absence of certainty? Certainty is commonly conceptualized as a subjective sense of confidence in the correctness of a belief or judgment (Fischhoff et al., 1977), and individuals generally strive for certainty to reduce ambiguity and stabilize their beliefs (Kruglanski and Webster, 1996). However, Pouget et al. (2016) argue that confidence and certainty are distinct cognitive constructs, with certainty representing a broader probabilistic computation that guides decision-making, and confidence referring specifically to the estimated likelihood that a particular judgment is correct. Extending this perspective, (Göken and Weger 2024) conceptualize certainty as a subjective experiential state comprising affective, cognitive, somatic, and behavioral components. As with uncertainty, certainty is therefore not a uniformly defined construct, but one that varies across individuals and contexts. A key methodological issue concerns how subjective uncertainty is measured. Two types of response scales are commonly used: (1) bipolar scales ranging from “absolutely certain” to “absolutely uncertain” (Lingam et al., 2025b), and (2) unipolar scales ranging from “not at all uncertain” to “absolutely uncertain” (Franssen et al., 2024a; Lingam et al., 2025a; Alquist et al., 2020). Bipolar scales assume that certainty and uncertainty are perfect inverses, implying mutual exclusivity. In contrast, unipolar scales treat uncertainty as an independent construct, allowing certainty and uncertainty to coexist or vary independently. At present, there is no consensus in the human factors literature regarding which approach is more appropriate. This uncertainty regarding measurement is reflected more broadly in the literature. Recent work has highlighted that uncertainty is conceptualized differently across fields such as neuroscience, robotics, and human-automation interaction, creating challenges for developing a common understanding of uncertainty-related constructs (Cooke et al., 2026). Furthermore, methodological research has shown that unipolar and bipolar scales cannot automatically be treated as interchangeable, as they may exhibit different measurement properties, response distributions, and underlying measurement structures (Höhne et al., 2022, 2021).

The central question of the present study is whether uncertainty reflects the absence of certainty, or whether certainty and uncertainty represent partially independent psychological dimensions. We hypothesize that these constructs are related, but not perfect inverses of one another. We posit that certainty and uncertainty may be experienced simultaneously as distinct psychological states, potentially creating cognitive dissonance. Consequently, their assessment requires separate unipolar measurement scales. To gather a very large group of diverse participants, we used a crowd-sourcing approach. This online crowd-sourced experimental study enables the recruitment of a broad demographic sample across various age groups and geographical regions (Crump et al., 2013; Hilbig, 2016), which has already been successfully implemented to study human factors in automotive contexts (Bazilinskyy et al., 2021; Oudshoorn et al., 2021). In order to induce conditions of potential uncertainty, we exposed participants to various driving situations around the occurrence of a proposed lane change maneuver. These situations were chosen because lane changes are considered one of the most dangerous driving maneuvers (Yan et al., 2023; Arefnezhad et al., 2018; Zheng et al., 2016).

## 2 Methods

Participants were presented online with 20 virtual driving scenarios with 3D animations. A set of 10 scenario videos was played twice, participants were asked to rate either their certainty or uncertainty about a specified maneuver and the opposite for the second time the videos were played, in a randomized order. The participants reviewed simulated dynamic scenarios (3D animations) that depict a mix of basic and slightly challenging traffic situations, like Figure 1 (more details are given in Section 2.3). The definition of the autonomy level was based on the Society of Automotive Engineers' (SAE) classifications: “Level 5 - Full driving automation” (SAE International, 2021).

**Figure 1:**
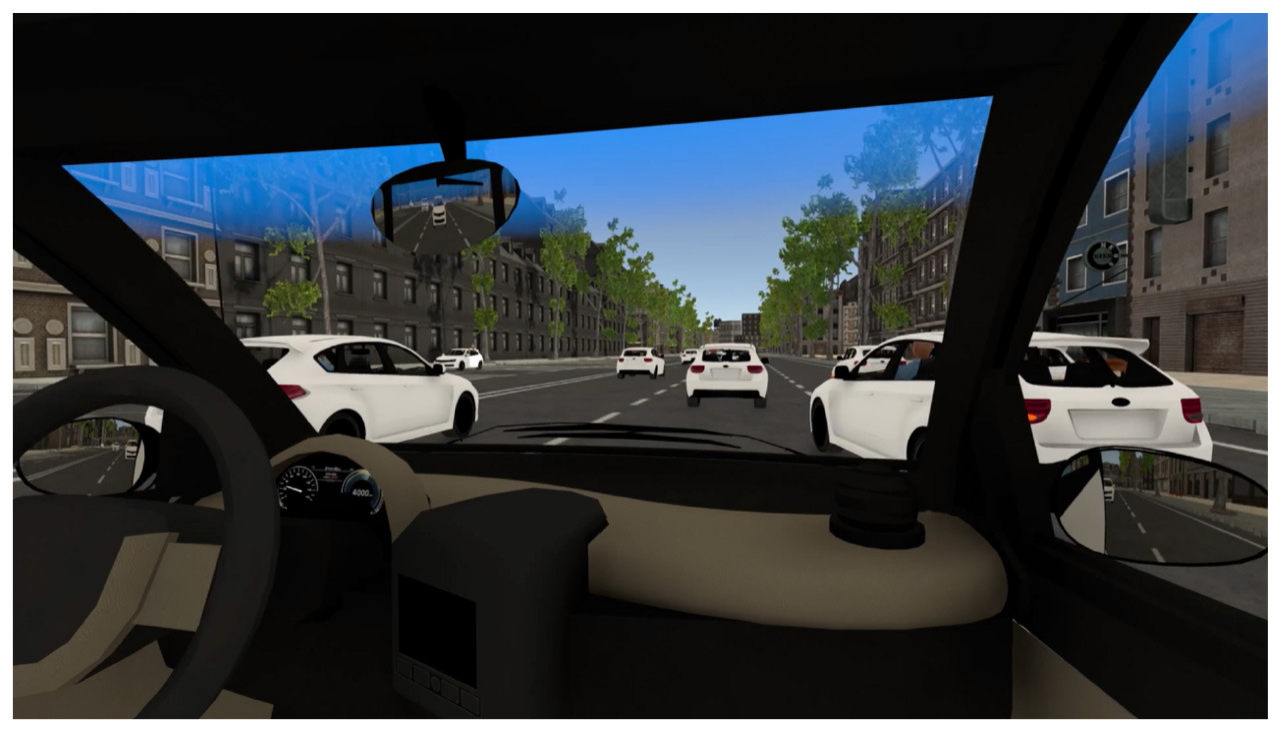
Scenario 8 fully automated car: a car overtakes you from the right lane.

### 2.1 Dependent measures

To study psychological certainty and uncertainty, we chose to let the participants use their own understanding of what they perceive as the feeling of certainty (perceived psychological certainty) and uncertainty (perceived psychological uncertainty), in order to measure it in its purest form as it would be experienced by passengers under those conditions. We clearly defined the action that the passenger could be (un)certain about, by asking: “*At the end of the scenario, the fully automated car changes to the left lane. How* ***(un)certain*** *would you feel?”*. This question was asked at the end of each scenario on the following screen (henceforth the scenario was not visible anymore). Perceived psychological certainty and uncertainty were measured using a subjective rating scale. After viewing each scenario, participants were instructed to use a slider scale ranging from “Absolutely not (un)certain” to “Absolutely (un)certain”. No numerical values were mentioned in order to minimize an anchoring bias (Lieder et al., 2018). The slider ranged from 0 (‘Absolutely not (un)certain”) to 100 (“Absolutely (un)certain”), keeping track of a numerical value in the back end. Similar approaches have been used successfully to measure the amount of uncertainty that users of a fully automated car experience based on the interior design of the car, in the work of Franssen et al. (2024a). Furthermore, Lingam et al. (2025a) use a similar approach in the context of autonomous drones using the phrases: “no uncertainty” to “absolute uncertainty”, as well as Alquist et al. (2020) who measure uncertainty on a scale from “not at all uncertain” to “very uncertain” in the context of executive function.

### 2.2 Participants

A total of 1,437 people (Valid N) (M = 705, F = 718, Other = 8, Prefer not to respond = 6) from 84 countries participated in the study. Because the objective of the study was to investigate the relationship between certainty and uncertainty rather than estimate prevalence within a specific national population, a heterogeneous international sample was considered advantageous. Recruiting participants from a large number of countries increased diversity in experiences and backgrounds, thereby supporting the generalizability of the observed measurement relationship across different populations. Initially, there were participants from 85 countries, but participants from Venezuela were removed after the researchers identified a pattern of cheating. All participants were 18 years or older (*M* = 35.55, *SD* = 11.00) and digitally provided their informed consent prior to participation. Participants were recruited through the crowd-sourcing service Appen ^1^ and were later redirected to Heroku ^2^ to review the stimuli.

The countries most represented were Brazil (*n* = 173), the United States (*n* = 125), India (*n* = 121) and the Philippines (*n* = 119). To prevent duplicate responses, participants were restricted to one submission per unique worker ID. A payment of 0.50 USD was offered after the completion of the experiment. This was in line with what similar crowd-sourced studies offered to their participants (Bazilinskyy et al., 2021; Oudshoorn et al., 2021).

The survey was awarded an overall satisfaction rating of 3.7 on a scale of 1 to 5 by 377 participants who completed the satisfaction survey. This study was approved by the Ethics Review Board of the Industrial Design Department at Eindhoven University of Technology (reference: ERB2023ID409).

### 2.3 Stimuli

We created 10 simulated 10-second videos recorded from the passenger seat of a fully automated car with no driver present (Figure 1) to compare uncertainty and certainty experienced by passengers of fully automated cars. These videos were presented in randomized order and twice, once in the context of certainty and once in the context of uncertainty, which was also randomized.

One challenge in using crowd-sourcing for scientific data collection is ensuring that participants fully understand the instructions. Long, complex instructions can cause participants to skim or overlook key details. To address this, we designed the instructions to be as brief and clear as possible. The participants were informed about this with the following instruction: *"During the experiment, the videos will show manually driven cars (cars with human drivers), except for the car that you are a passenger in. The car that you are a passenger in is a fully automated car (a robotaxi that you are a passenger in). The fully automated car controls all driving-related tasks without input from a human driver. The fully automated car is displayed with a blue overlay on the windows."*

The type of scenarios can be found in Table 1. Each scenario took place on a six-lane road. Three lanes for the participant's direction and three lanes for oncoming traffic, as can be seen in Figure 1. A double uninterrupted road marking was used to divide the sets of three lanes with opposing traffic. The participant's car was always in the middle lane and did not change lanes during the scenario. The scenario ended just before the car initiated a lane change to the left, which was not visible in the video.

**Table 1:** Scenario description.

Scenario	Depicted scenario	Safe to change to left lane	Speed (km/h)
1	A car has just overtaken you from the left lane	Yes	30
2	Basic traffic	Yes	30
3	A car is approaching in the left lane	Debatable	30
4	A car in the left lane is overtaking you	No	30
5	A car behind is tailgating	Yes	30
6	Basic traffic	Yes	30
7	A car in the right lane wants to overtake you	Yes	30
8	A car overtakes you from the right lane	Debatable	30
9	A car approaches you from the left lane	Debatable	60
10	A car overtakes you from the left lane, and a second car is about to overtake you	No	30

All scenarios were designed to be neutral or mildly provoking based on the expert researcher's judgment. For an overview of the scenarios, see Table 1. The provocation scenarios included unusual behavior by other road users such as overtaking from the right (Figure 1), or other unusual behavior. The provoking scenarios are based on elements of the Driving Behavior Questionnaire (DBQ) (Lajunen et al., 2004; Martinussen et al., 2013), the Positive Behavioral Questionnaire (PBQ) (Özkan and Lajunen, 2005), and the Driver Anger Scale (DAS) (Deffenbacher et al., 1994). We ran a pilot study with five participants to improve the stimuli, scenarios, and procedure. The pilot study first contained 5-second video scenarios. From the pilot study, we learned that 5-second videos were too short to experience uncertainty, however longer videos beyond 10 seconds led to a loss of focus. Based on this, we opted for a 10-second video length.

To maintain a high degree of experimental control and minimize unintended variation between conditions, all scenarios were created in Unity, ensuring a standardized environment across the scenarios. The simulation did not include motion blur, which can indicate speed (Lin and Li, 2004). However, research by Langbehn et al. (2016) indicates that motion blur does not have a noticeable effect on how humans perceive speed in simulations. The cars always drove 30 km/h, except in Scenario 9, where all the cars drove at a speed of 60 km/h. The higher speed was introduced as a potential uncertainty-provoking element. As seen in Figure 1, the car always drove in the middle lane of a three-lane road. Following each video, participants were asked to either rate their certainty or uncertainty about the upcoming lane change. The phrasing was standardized to ensure comparability between conditions:

- After each video of the fully automated car, we asked: “*At the end of the scenario the fully automated car changes to the left lane. How* ***uncertain*** *would you feel?”*.
- After each video of the fully automated car, we asked: “*At the end of the scenario the fully automated car changes to the left lane. How* ***certain*** *would you feel?”*

### 2.4 Procedure

The study was presented in English. Contact information was provided at the top of the website introducing this study. Participants were informed that they could contact the researchers to ask questions about the study and that they had to be at least 18 years of age. Information about anonymity and the voluntary nature of participation was also provided. The participants first provided demographic information. The participants were then asked to leave the questionnaire by clicking on a link that opened a (Heroku) website with the experiment. The Heroku platform contained the randomly shuffled stimuli (animations), which lasted 10 s, that were followed up with the (un)certainty slider question which is elaborated on in the 2.1 subsection. Data quality assurance measures are taken to ensure attention and filter for invalid data. Participants must move the slider before they can continue. This ensures that participants cannot skip slider inputs and are unable to continuously click through all inputs without giving a real input.

### 2.5 Data analysis

To examine the relationship between certainty and uncertainty, a linear mixed-effects model (LMM) was conducted. Model convergence and covariance parameter estimates were inspected as part of model evaluation. This approach was chosen to appropriately account for the hierarchical structure of the data, in which multiple observations (video evaluations) were nested within participants. Certainty scores were included as a continuous fixed effect predictor, and uncertainty scores served as the dependent variable. To account for variability between individuals, random intercepts were specified for participants. Additionally, repeated measurements across scenarios were modeled using a repeated covariance structure (diagonal), allowing for variation in residuals across stimuli while accounting for within-subject dependencies. The use of a mixed-effects modeling approach was necessary because each participant evaluated multiple videos, resulting in non-independent observations. Linear mixed models are often fairly robust to violations of normality assumptions in the observed variables and provide more accurate parameter estimates and standard errors under such conditions (Schielzeth et al., 2020). Model parameters were estimated using maximum likelihood estimation. Statistical significance of fixed effects was evaluated using F-tests, with degrees of freedom approximated using the Satterthwaite method. In addition to significance testing, the unstandardized regression coefficient (B) and its 95% confidence interval were examined to assess the strength and nature of the relationship between certainty and uncertainty. All analyses were conducted in IBM SPSS Statistics (Hinton et al., 2014) using the Linear Mixed Models procedure. As a post-hoc robustness check, the primary linear mixed-effects model was repeated using only participants from predominantly English-speaking countries (United States, United Kingdom, Canada, Australia, Ireland, and New Zealand) to examine whether the findings could be explained by differences in English language proficiency.

## 3 Findings

### 3.1 Uncertainty is not the inverse of certainty

A linear mixed-effects model was conducted to examine the relationship between certainty and uncertainty, while accounting for repeated measurements within participants and variability across scenarios. Certainty was included as a fixed effect, with random intercepts specified for participants and repeated measurements across videos. The analysis revealed a significant negative effect of certainty on uncertainty, F_(1, 14353.89)_ = 1610.83, *p* < 0.01. The estimated regression coefficient indicated that higher levels of certainty were associated with lower levels of uncertainty (*B* = −0.320, 95% CI [−0.335, −0.304]). This coefficient suggests that for every 10-point increase in certainty, uncertainty decreased by approximately 3.2 points. While this indicates a clear inverse relationship between the two variables, the magnitude of this effect is substantially smaller than what would be expected if uncertainty were simply the absence of certainty (i.e., *B* = −1). These findings demonstrate that certainty and uncertainty are not perfectly complementary constructs. Instead, they appear to represent partially independent dimensions, such that increases in certainty are associated with decreases in uncertainty, but not in a one-to-one inverse manner. Furthermore, the 95% confidence interval around the regression coefficient did not include −1, providing additional evidence against the assumption of a perfectly inverse relationship. Figures 2 and 3 depict the distributions of uncertainty and certainty ratings across the ten driving scenarios. Considerable variability was observed within each scenario, as reflected by the broad interquartile ranges and the wide spread of responses across the rating scale. This indicates that responses were not restricted to a narrow range of values.

**Figure 2:**
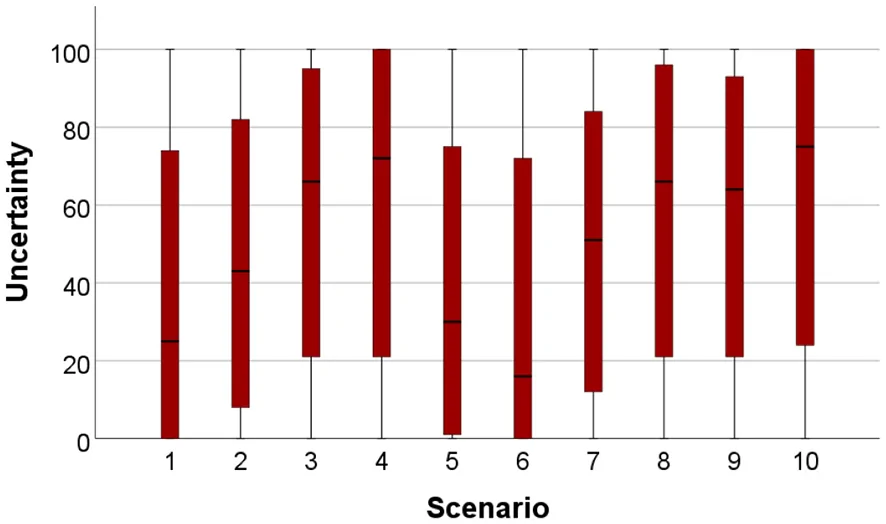
Boxplot of uncertainty values per scenario.

**Figure 3:**
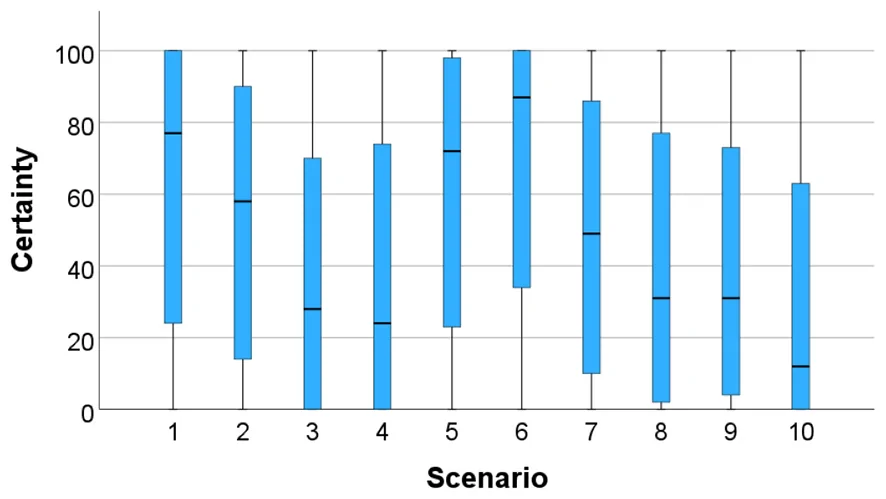
Boxplot of certainty values per scenario.

### 3.2 Descriptive statistics and response variability

Descriptive statistics for certainty and uncertainty ratings across the 10 scenarios are presented in Table 2. Across all scenarios, participants used the full response scale (0–100) for both certainty and uncertainty ratings. Furthermore, substantial variability was observed within each scenario, with standard deviations ranging from 36.03 to 38.93 for certainty and from 36.78 to 38.88 for uncertainty. Mean certainty ratings varied from 31.48 to 67.30 across scenarios, whereas mean uncertainty ratings varied from 34.43 to 62.43. Together, these findings indicate considerable variability in responses and suggest that the observed relationship between certainty and uncertainty was unlikely to be driven by restricted response variability.

**Table 2:** Descriptive statistics for certainty and uncertainty ratings across the ten scenarios.

Scenario	Mean certainty	SD certainty	Mean uncertainty	SD uncertainty
1	61.83	38.93	37.93	37.92
2	52.38	37.62	46.69	37.40
3	37.78	36.03	57.21	37.25
4	37.48	37.94	60.47	38.52
5	60.03	38.06	40.61	37.79
6	67.30	38.44	34.43	38.88
7	48.87	37.48	49.28	36.89
8	41.26	37.21	57.12	37.40
9	40.67	36.09	56.12	36.78
10	31.48	36.83	62.43	38.57

### 3.3 Sensitivity analysis: English-speaking countries

To assess whether the observed relationship could be explained by variation in English language proficiency, the primary model was repeated using only participants from predominantly English-speaking countries (United States, United Kingdom, Canada, Australia, Ireland, and New Zealand). The relationship between certainty and uncertainty remained significant, F_(1, 2408)_ = 556.57, *p* < .001. Higher certainty was associated with lower uncertainty (*B* = −0.432, 95% CI [−0.468, −0.396]). The persistence of the effect within this subsample suggests that the observed relationship was not dependent on the inclusion of participants from non-English-speaking countries. Importantly, the estimated coefficient remained far from the value that would be expected if uncertainty merely reflected the absence of certainty (*B* = −1).

## 4 Discussion

### 4.1 Interpretation of the findings

The present study examined whether uncertainty can be conceptualized as the simple absence or inverse of certainty. Using a linear mixed-effects approach that accounted for repeated measurements within participants and variability across stimuli, a significant negative relationship between certainty and uncertainty was observed. However, crucially, the magnitude of this relationship (*B* = −0.320) deviated substantially from a perfect inverse association (*B* = −1). This suggests that, although certainty and uncertainty are related, they cannot be treated as direct opposites. Instead, the results support the interpretation that certainty and uncertainty represent partially independent psychological constructs. In practical terms, this means that increases in certainty do not fully eliminate uncertainty, and conversely, low certainty does not imply maximal uncertainty. This challenges simplified conceptualizations in which the two dimensions are assumed to lie on a single continuum. An interpretation of these findings is that individuals can simultaneously experience elements of both certainty and uncertainty. For example, a person may feel relatively confident about one aspect of a stimulus while still experiencing doubt about another. This implies the use of unipolar scales to measure perceived psychological certainty and uncertainty as demonstrated in the works of Franssen et al. (2024a) Lingam et al. (2025a) and Alquist et al. (2020).

### 4.2 Methodological considerations and robustness

The use of a multilevel modeling approach strengthens the validity of these conclusions. By accounting for the nested structure of the data (multiple scenario evaluations per participant), the analysis avoids biases associated with non-independent observations and provides a more accurate estimate of the relationship between certainty and uncertainty. Additionally, incorporating variability across stimuli ensures that the observed effects are not driven by specific scenario characteristics. The robustness of the findings is further supported by several observations. The model converged successfully, and the covariance parameter estimates supported the inclusion of participant-level random intercepts. In addition, substantial variability was observed across certainty and uncertainty ratings, reducing concerns that the observed relationship resulted from restricted response ranges. Finally, a sensitivity analysis restricted to participants from predominantly English-speaking countries yielded the same substantive conclusion as the primary analysis, suggesting that the findings were not dependent on the linguistic diversity of the sample. Together, these observations indicate that the reported deviation from a perfect inverse relationship cannot readily be attributed to restricted response variability or language-related differences between participants.

### 4.3 Practical and research implications

The present findings have implications for both research and the design of automated driving systems. From a measurement perspective, the results suggest that certainty and uncertainty should not automatically be treated as opposite ends of a single dimension. Researchers using bipolar certainty-uncertainty scales should be aware that such measures implicitly assume a perfect inverse relationship between the two constructs, an assumption that was not supported by the present findings. From an applied perspective, the findings have implications for human-automation interaction. Psychological uncertainty has previously been associated with trust in automated systems, whereas trust has been identified as a key determinant of automated vehicle acceptance (Lee and See, 2004; Hoff and Bashir, 2015; Choi and Ji, 2015). Moreover, trust is increasingly recognized as a central construct in automated driving research, influencing user interaction, reliance, and acceptance of automated systems (Walker et al., 2023). If certainty and uncertainty are not direct opposites, interventions aimed at reducing uncertainty may not automatically increase certainty to the same extent. Designers of automated driving systems should therefore consider assessing both constructs separately when evaluating transparency features, communication strategies, explanations, or interface designs. For example, an interface feature that reduces passenger uncertainty may not necessarily produce an equivalent increase in passenger certainty. More broadly, a more nuanced understanding of certainty and uncertainty may contribute to more accurate evaluations of trust, user experience, and acceptance of automated driving systems. If certainty and uncertainty contribute differently to these outcomes, relying on a single bipolar measure may obscure important distinctions. Future research could investigate whether certainty and uncertainty make independent contributions to these outcomes and whether they should therefore be considered distinct constructs in human factors research.

### 4.4 Limitations

Both certainty and uncertainty were measured using subjective self-report scales, which may be influenced by individual response tendencies or interpretation of the scale. Second, although the use of multiple videos increases generalizability across stimuli, the specific content of these videos was not systematically manipulated, limiting conclusions about causal drivers of certainty and uncertainty. Future research could address this by experimentally varying stimulus ambiguity or informational clarity. Third, although the study included participants from 84 countries, it was not designed to investigate cultural differences in certainty and uncertainty. The international sample was intentionally used to increase heterogeneity and support the generalizability of the observed measurement relationship. However, cultural or regional variations in the relationship between certainty and uncertainty were beyond the scope of the present study and could be examined in future research.

### 4.5 Future work

Future research could further investigate the relationship between certainty and uncertainty by examining potential variability across individuals and contexts. For instance, it would be valuable to explore whether some individuals exhibit a stronger inverse relationship between certainty and uncertainty than others, potentially reflecting differences in cognitive style, tolerance for ambiguity, or metacognitive awareness. Furthermore, the link between uncertainty and trust as discussed by Lingam et al. (2026) could further be complemented by separately measuring the effects that certainty has on trust and vice-versa. Future research could investigate whether certainty and uncertainty make independent contributions to trust, user experience, and acceptance, or whether these effects are primarily driven by one of the two constructs.

In addition, future studies could systematically manipulate characteristics of the stimuli, such as ambiguity, complexity, or informational clarity, to better understand the conditions under which certainty and uncertainty diverge. This would allow for more precise conclusions about the mechanisms underlying both constructs, rather than relying on naturally varying stimuli as in the present study. Another promising direction would be to extend the measurement approach. While the current study relied on self-reported ratings, incorporating behavioral or physiological measures (e.g., response times, eye-tracking, or neural indicators) could provide additional insight into how certainty and uncertainty are experienced and processed. This multimethod approach may help determine whether the observed partial independence generalizes across different levels of measurement.

Finally, future work could employ more advanced modeling approaches, such as random slopes in multilevel models, to examine whether the relationship between certainty and uncertainty varies systematically across participants or stimuli. This would deepen our understanding of the extent to which the observed relationship reflects a general psychological principle versus context-specific dynamics.

## 5 Conclusion

The present findings provide empirical evidence that certainty and uncertainty are not simply opposing ends of a single dimension. Instead, they appear to function as related but distinct constructs. The present findings indicate that psychological certainty and uncertainty can coexist and should therefore be assessed using separate unipolar scales rather than a single bipolar scale. Furthermore, this offers new insights for theoretical models and shows that uncertainty cannot merely be treated as the absence of certainty, and suggests that more nuanced conceptualizations are needed to accurately capture how individuals evaluate and respond to ambiguous information.

## Data Availability

The datasets presented in this study can be found in online repositories. The names of the repository/repositories and accession number(s) can be found below: https://doi.org/10.4121/b61ddc8f-0c07-44b5-b570-6208edc2f4cb.

## Footnotes

1
https://appen.com

2
https://www.heroku.com
